# High mortality among hospitalized adult patients with COVID-19 pneumonia in Peru: A single centre retrospective cohort study

**DOI:** 10.1371/journal.pone.0265089

**Published:** 2022-03-08

**Authors:** Guiliana Mas-Ubillus, Pedro J. Ortiz, Jorge Huaringa-Marcelo, Paola Sarzo-Miranda, Patricia Muñoz-Aguirre, Alejandra Diaz-Ramos, Kattia Arribasplata-Purizaca, Doris Mendoza, Juan Rojas-Poma, Cristian Marcelo-Ruiz, Pedro Ayala-Diaz, Edwin Hidalgo-Arroyo, Lourdes Tupia-Cespedes

**Affiliations:** 1 Department of Emergency, Hospital Nacional Arzobispo Loayza, Cercado de Lima, Lima, Peru; 2 Facultad de Medicina, Universidad Peruana Cayetano Heredia, San Martín de Porres, Lima, Peru; 3 Facultad de Medicina, Universidad Científica del Sur. Carr, Villa El Salvador, Lima, Peru; SGPGIMS: Sanjay Gandhi Post Graduate Institute of Medical Sciences, INDIA

## Abstract

**Background:**

Peru is the country with the world’s highest COVID-19 death rate per capita. Characteristics associated with increased mortality among adult patients with COVID-19 pneumonia in this setting are not well described.

**Methods:**

Retrospective, single-center cohort study including 1537 adult patients hospitalized with a diagnosis of SARS-CoV-2 pneumonia between May 2020 and August 2020 at a national hospital in Lima, Peru. The primary outcome measure was in-hospital mortality.

**Results:**

In-hospital mortality was 49.71%. The mean age was 60 ± 14.25 years, and 68.38% were males. We found an association between mortality and inflammatory markers, mainly leukocytes, D-dimer, lactate dehydrogenase, C-reactive protein and ferritin. A multivariate model adjusted for age, hypertension, diabetes mellitus, and corticosteroid use demonstrated that in-hospital mortality was associated with greater age (RR: 2.01, 95%CI: 1.59–2.52) and a higher level of oxygen requirement (RR: 2.77, 95%CI: 2.13–3.62). Conclusions: In-hospital mortality among COVID-19 patients in Peru is high and is associated with greater age and higher oxygen requirements.

## Introduction

After the emergence of severe acute respiratory syndrome coronavirus type 2 (SARS-CoV-2) infection in Wuhan, China, the disease rapidly spread across other countries [1] leading to the declaration of a novel coronavirus disease (COVID-19) pandemic by the World Health Organization on January 11, 2020 [2]. To date, during the remission phase of the second wave of infection in Peru, there were 2,007,477 confirmed cases and 189,261 deaths, presenting the world’s highest death rate per capita [3–5], see S1 Graph and S1 Table.

Several studies have demonstrated that age, obesity, diabetes mellitus, hypertension, and kidney disease are risk factors associated with the development of severe complications by SARS-CoV2 [6–8]. There is also evidence of its association with certain laboratory findings that are comprised in severity risk scores [9–11]. Furthermore, the association between mortality and low oxygen saturation is another finding that has been well documented elsewhere and demonstrated in our population [12].

These findings can be explained by delayed access to health care that is reflected in severe disease cases. This delay is largely the result of deficiencies in our primary care system, the lack of molecular tests and their replacement by non-reliable serological tests, self-medication and home management due to the overload of health care capacity, and the shortage of intensive care unit (ICU) beds for providing advanced support [13–15].

To date, COVID-19 is still a highly fatal health care concern given its high transmissibility, delayed vaccine implementation, and the social-sanitary problems existing in our country [12,16], and the disease remains challenging due to the possibility of consecutive waves of infection in the future. Hence, the identification of the risk factors for developing severe disease has a key role in controlling the infection, in health education for the general population, and in the implementation of public health measures.

The aim of this study was to determine the characteristics associated with high mortality in adult patients diagnosed with SARS-CoV-2 pneumonia in a tertiary care hospital during the health care emergency in Lima, Peru.

## Materials and methods

### Study design and setting

A retrospective cohort study was carried out at the *Hospital Nacional Arzobispo Loayza* (HNAL), a national referral hospital located in Lima, the capital of Peru. The hospital has approximately 700 beds for the emergency department, hospitalization wards, and intensive care unit (ICU) [17]. During the SARS-CoV-2 pandemic, many hospital wards were dedicated to only COVID-19 pneumonia care, until bed capacity was exceeded and additional hospitalization beds were added to open areas of the hospital [18].

### Data source and patient population

Data were extracted from paper medical records revised at patient discharge, death, or transfer. We included data from all adult patients aged ≥ 18 years with either suspected (compatible signs and symptoms with suggestive laboratory or radiological findings and with or without a negative rapid serological test) or confirmed (same findings but with a positive rapid serological or in RT-PCR test) diagnosis of SARS-CoV-2 pneumonia, admitted to the HNAL between May 1 to August 31, 2020. Due to the nationwide shortage of diagnostic tests during the study period [19], symptomatic patients were admitted as suspected cases due to the clinical characteristics and supported by typical laboratory markers and radiological findings as established by national guidelines without a diagnostic test [20,21]. Study size was the amount of medical records collected.

We excluded patients with mild SARS-CoV-2 infection discharged for outpatient management; a mild case was defined by a patient with symptoms of COVID-19 but without dyspnea or radiologic findings suggestive of pneumonia and who has a SO2 higher than 95% while breathing ambient air, as the Peruvian National Guidelines of COVID-19 management stated [21]. We also excluded patients who left the hospital against medical advice within 24 hours of admission, patients without respiratory symptoms and signs, mainly gastrointestinal and surgical conditions admitted with a positive serological test, and patients who died within a short time after admission to collect enough data to be included.

### Data collection

The patient information collected included: demographic data, COVID-19 test results, duration of symptoms before hospital admission, signs and symptoms, oxygen saturation, fraction of inspired oxygen, oxygen delivery device, comorbidities, self-medication, laboratory findings, and medical treatment received during hospitalization. All laboratory tests were processed in the laboratory of the HNAL.

The level of oxygen requirement was classified into three categories depending on three criteria evaluated on admission to the emergency department: arterial oxygen partial pressure to fractional inspired oxygen (PaO2/FiO2) ratio, oxygen delivery, and oxygen saturation (SaO2) with FiO2 of 21%. When the PaO2/FiO2 ratio was not available, it was estimated from the SaO2/FiO2 ratio using the Ellis-Severinghause formula [22,23]. According to these criteria, patients were classified in the first level if they had PaO2/FiO2 ratio >300 mmHg, need for oxygen delivery at a maximum of 2 liters per minute (28% FiO2) through a nasal cannula, or SaO2 >90% with FiO2 of 21%. The second level was defined as a PaO2/FiO2 ratio between 150 to 300 mmHg, need for oxygen delivery at a maximum of 5 liters (40% FiO2) through a nasal cannula, or SaO2 between 80–90% with FiO2 of 21%. Finally, the third level was defined as a PaO2/FiO2 ratio <150 mmHg, need of oxygen delivery with FiO2 >40% or through device other than a nasal cannula, or SaO2 <80% with FiO2 of 21%; see S2 Table.

### Study outcomes

The main outcome was in-hospital mortality. In-hospital mortality was determined by verifying medical records, patient condition at discharge in the HNAL computerized system, and confirming the date and hour of death in the Peruvian National Death Information System (SINADEF) [24] according to names, last names, and national identity number.

### Statistical analysis

Categorical variables were reported as frequencies and percentages (%), and continuous variables were expressed as mean and standard deviation or medians with interquartile range (IQR) according to variable distribution. For exploratory analysis, we used the chi-squared test or Fisher’s exact test to compare dichotomic variables. The Student’s t-test was performed to compare one categorical and another continuous variable for normally distributed data; otherwise, the Mann-Whitney U test was applied. To compare polytomous and continuous variables, the ANOVA test or Kruskal Wallis test was performed depending on the statistical distribution. Finally, a multivariate regression analysis was conducted using the Poisson model with robust variance to determine the effect of each covariate on mortality, adjusting for known confounding variables. We considered comorbidities such as diabetes mellitus, hypertension, obesity, and corticoid use during hospitalization as possible confounding variables. We considered a confidence level of 95% and a p-value <0.05 as statistically significant. The statistical analyses were performed using Stata software version 14.2.

### Ethical statement

The study was approved by the Institutional Ethics Committee for Research at the Hospital Nacional Arzobispo Loayza in Lima, Peru (Reference number: 011–2021) with waiver of patient consent on the basis of study design and the ongoing COVID-19 public health emergency.

## Results

### Baseline characteristics and patient outcomes

**Fig 1:**
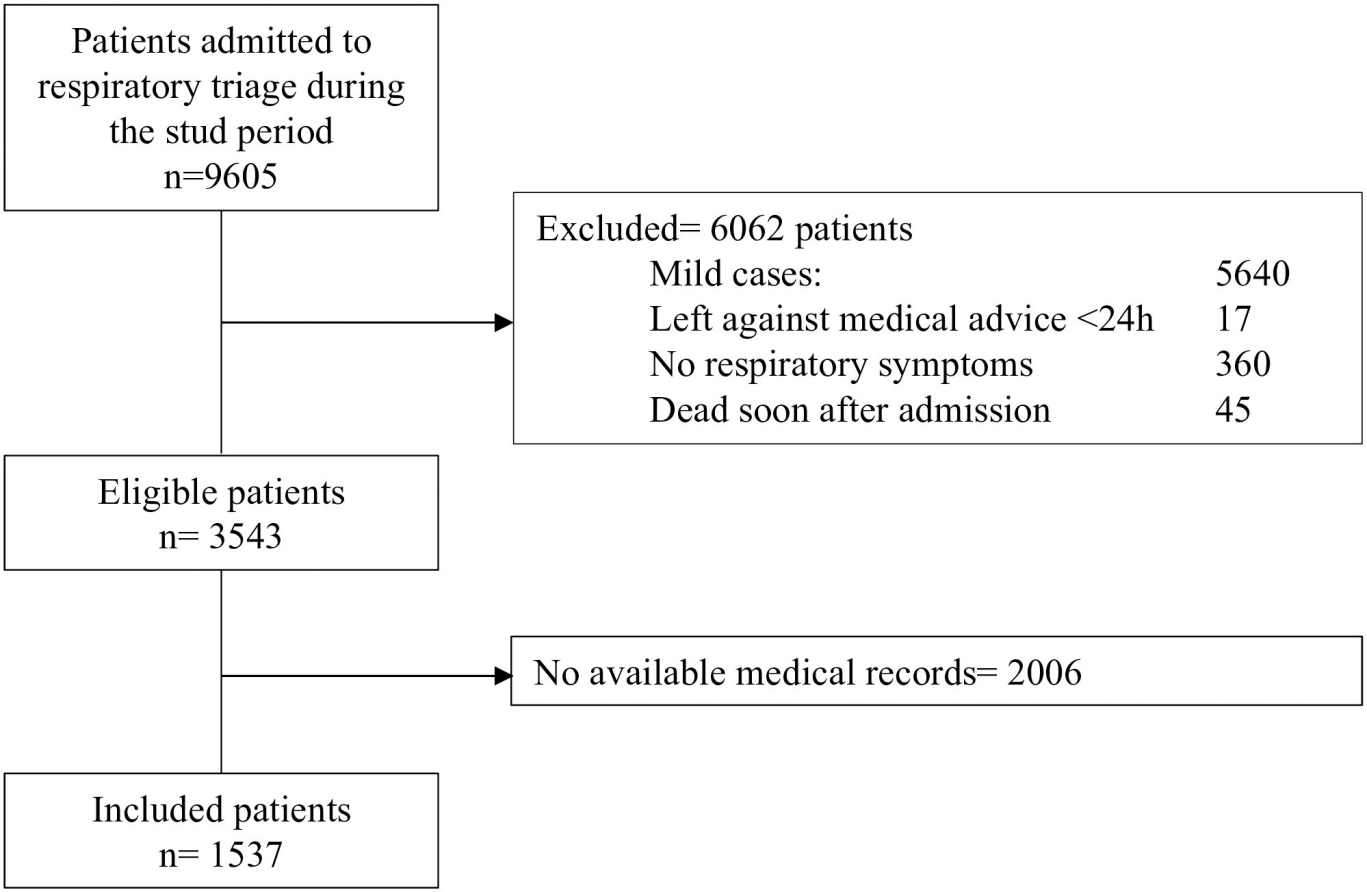
Flowchart of participants.

From May 1 to August 31, 2020, 9605 patients with either suspected or confirmed diagnosis of SARS-CoV-2 infection were admitted to the emergency department. Among these patients, 6062 were excluded because they did not meet the inclusion criteria. Of the 3543 potentially eligible patients, 2006 medical records were not accessible because they were stored by the Administration Office to ensure biosecurity. Finally, 1537 patients were included in the study. Fig 1.

We classified 1097 patients as definitive cases (71.4%); however, due to the lack of definitive test 440 patients were classified as suspected cases (28.6%) and only 39 patients had a RT-PCR test done. Among the 1537 patients included in the study, 68.4% were males and 31.6% females with a mean age of 60 ± 14.25 years. The median length of symptom duration was 7 days (IQR: 5–10). Dyspnea was the most frequent symptom (85.1%), followed by cough (68%), malaise (53.5%), and fever (48.4%). The most common comorbidities were hypertension in 26.3% of cases and type 2 diabetes mellitus in 18.7%. Slightly over one quarter of the patients reported having received some medication (corticosteroids, antibiotics, or anticoagulants) before hospitalization. During hospital stay, 98.35% of patients received antibiotic therapy, whereas 89.93% received some type of corticosteroids such as methylprednisolone, prednisone, dexamethasone, and hydrocortisone, at variable doses ranging from low to corticosteroid pulses. The median length of hospital stay was 7.5 days (IQR: 2.4–14.5) and the general in-hospital mortality was 49.71% (764 patients) Table 1.

**Table 1:** Characteristics of hospitalized patients with SARS-CoV-2 pneumonia.

Variables	
Age in years^a^	60.1 ± 14.3
Male gender	1051 (68.4)
Duration of symptoms in days^b^	7 (5–10)
Fever	727 (48.4)
Cough	1022 (67.9)
Dyspnea	1289 (85.1)
Sore throat	338 (22.5)
Malaise	804 (53.5)
Chest pain	97 (6.5)
Headache	116 (7.7)
Vomiting	40 (2.7)
Diarrhea	42 (2.8)
Hypertension	401 (26.3)
Diabetes Mellitus	286 (18.7)
Obesity	241 (15.8)
Chronic kidney disease	52 (3.4)
Malignant neoplasm	14 (0.9)
Human immunodeficiency virus	16 (1.1)
Self-medication	402 (28.9)
Antibiotic use during hospitalization	1486 (98.3)
Corticosteroid use during hospitalization	1346 (89.4)
Length of hospital stay in days^b^	7.5 (2.4 a 14.5)
Mortality	764 (49.7)

Values are in number and percentage (%).

^a^Mean ± standard deviation.

^b^Median (interquartile range).

### Univariate analysis

Mortality was similar in both genders (p = 0.464). There was a higher frequency of deaths among patients aged 45–65 years and especially in those over 65 years of age, compared to patients less than 45 years old (p<0.001). Hypertensive patients had a greater mortality than non-hypertensive patients (p = 0.003). Regarding laboratory tests, there was an association between mortality and the majority of inflammatory markers. Patients who met the criteria for levels 3 of oxygen requirement on admission presented a higher mortality than those classified as level 1 Table 2.

**Table 2:** Association between mortality and main covariates.

Variables	Discharged n = 773	Deceased n = 764	P value
Male gender	536 (69.3)	515 (67.4)	0.464
Age in years^a^	55.8 ± 13.3	64.5 ± 13.8	< 0.001
Diabetes	136 (17.6)	150 (19.6)	0.325
Hypertension	176 (22.8)	225 (29.5)	0.003
Obesity	125 (16.2)	116 (15.2)	0.624
Antibiotic use during hospitalization	742 (95.9)	744 (97.4)	0.105
Corticosteroid use during hospitalization	654 (84.6)	692 (90.6)	<0.001
Duration of symptoms in days^b^	7 (4–10)	7 (5–10)	0.002
Length of hospital stay in days^b^	4.5 (1–10)	10.9 (5–18)	<0.001
Oxygen requirement level 3	180 (23.3)	501 (65.6)	< 0.001
Leukocytes ≥ 10000 (per mm3)	379 (49)	486 (63.6)	< 0.001
Neutrophils ≥ 7500 (per mm3)	452 (58.5)	537 (70.3)	< 0.001
Lymphocytes < 1000 (per mm3)	379 (49)	443 (57.9)	< 0.001
Platelets < 200 000 (per mm3)	69 (8.9)	121 (15.8)	< 0.001
D-dimer ≥ 1.0 (ug/ml)	71 (9.2)	113 (14.8)	< 0.001
Lactate dehydrogenase ≥ 350 (U/L)	334 (43.2)	513 (67.1)	< 0.001
C-reactive protein > 15 (mg/L)	270 (34.9)	320 (41.9)	< 0.001
Glucose > 180 (mg/dl)	154 (19.9)	204 (26.7)	< 0.001
Lactate ≥ 2 (mmol/L)	83 (10.7)	152 (19.9)	< 0.001
Fibrinogen < 400 (mg/dl)	60 (7.8)	84 (10.9)	0.005
Creatinine ≥ 1 (mg/dl)	122 (15.8)	233 (30.5)	< 0.001
Alanine aminotransferase ≥ 35 (UI/L)	471 (60.9)	404 (52.9)	0.004
Total bilirubin ≥ 1 (mg/dl)	55 (7.1)	85 (11.1)	0.013
Albumin < 3.5 (mg/dl)	170 (21.9)	233 (30.5)	< 0.001
Troponin ≥ 0.001 (ng/L)	13 (1.7)	35 (4.6)	< 0.001
Procalcitonin ≥ 0.25 (ng/L)	17 (2.2)	29 (3.8)	0.042
Ferritin ≥ 1500 (ug/L)	79 (10.2)	105 (13.7)	0.003

Values are in number and percentage (%).

^a^Mean ± standard deviation.

^b^Median (interquartile range).

Patients of older age (63 years ± 13.6) presented higher oxygen requirements on hospital admission. Regarding the remaining covariates, we found that level 3 of oxygen requirement was associated with higher values of leukocytes, neutrophils, D-dimer, lactate dehydrogenase, C-reactive protein, lactate, and ferritin; and with lower values of lymphocytes and albumin. See S3 Table.

### Multivariate analysis

Higher levels of oxygen requirement and advanced age were associated with elevated in-hospital mortality. The analysis was adjusted for age, gender, hypertension, diabetes mellitus, obesity, and corticoid use during hospital stay. Patients with level 3 of oxygen requirement were nearly three times more likely to die than patients in level 1 (relative ratio [RR]: 2.77, 95% confidence interval [CI]: 2.13–3.62). In addition, patients in level 2 had a 33% greater probability of death than those in level 1 (RR: 1.33, 95% CI: 1.01–1.75), and this difference was statistically significant. We also found that patients over 65 years old had a two-fold greater likelihood of death than patients under 45 years of age (RR: 2.01, 95% CI: 1.59–2.52). Furthermore, patients between 45–65 years of age had a 51% greater probability to die than those under 45 years of age, and this association was statistically significant (RR: 1.51, 95% CI: 1.20–1.90) Table 3.

**Table 3:** Factors independently associated with mortality in multivariate analysis.

	Crude model		Adjusted model^a^	
Variables	RR (95% CI)	p-value	RR	p-value
Oxygen requirement level				
Level 1 (Low)	(ref)		(ref)	
Level 2 (Moderate)	1.43 (1.08–1.89)	0.012	1.33 (1.01–1.75)	0.047
Level 3 (High)	3.17 (2.46–4.11)	< 0.001	2.77 (2.13–3.62)	< 0.001
Age in years				
18–45	(ref)	.	(ref)	
45–65	1.72 (1.35–2.20)	< 0.001	1.51 (1.20–1.90)	< 0.001
> 65	2.56 (2.02–3.24)	< 0.001	2.01 (1.59–2.52)	< 0.001
Gender				
Female	(ref)		(ref)	
Male	0.96 (0.86–1.07)	0.412	0.99 (0.90–1.09)	0.848
Hypertension				
No	(ref)		(ref)	
Yes	1.18 (1.06–1.32)	0.002	1.04 (0.94–1.15)	0.41
Diabetes Mellitus				
No	(ref)		(ref)	
Yes	1.07 (0.94–1.21)	0.288	1.01 (0.90–1.15)	0.749
Obesity				
No	(ref)		(ref)	
Yes	0.96 (0.84–1.11)	0.607	1.02 (0.90–1.16)	0.733
Corticosteroid use during hospitalization				
No	(ref)		(ref)	
Yes	1.44 (1.16–1.79)	0.001	1.2 (0.98–1.47)	0.077

RR: Relative ratio; CI: Confidence interval.

^a^Model adjusted for age, gender, hypertension, diabetes, obesity.

## Discussion

In our study, we found an in-hospital mortality of about 50%, which is consistent with a previous report from Peru [12] and is among the highest mortality rates documented in the literature. The highest in-hospital mortality rate reported was 29.7% in Italy [25] followed by United States of America with 21.5% [16,26]. Similar rates have only been reported in patients hospitalized in critical-care units [16]. Our mortality rate is in line with the high death rate reported in Latin American countries, and to date, Peru has more than doubled its COVID-19 death toll following a data review, making it the country with the world’s highest death rate per capita [3,27].

Peruvian health system has particular characteristics that differ from other countries. First, healthcare system is fragmented, so different entities—from public to particular—deliver care in different and independent ways between each other [28]; the number of ICU beds were the lowest comparing to other Latin-American countries such as Colombia, Argentina and Brazil (4); oxygen supply was scarce, placed the country in the so named “the oxygen crisis” with oxygen plants that doesn’t work or can’t produce enough [29,30]; and finally, the number of trained health professionals in ICU care is scarce and mostly concentrated in Lima, the capital of Peru (4).

Although clinical and laboratory factors associated with mortality have been well documented [31] and prognostic scales have been developed for certain groups [10,11], our study demonstrates that age and low oxygen saturation on admission are proportionally associated with mortality. Traditional risk factors described in the literature could be applied to our population; however, in our analysis, these associations could be hidden due to the predominance of severe cases with significant respiratory compromise. Although this could be explained by the phenomenon of “silent hypoxemia” [32] which can delay health care-seeking, it does reflect a delay but in much more advanced and severe stages of the disease [5,12].

The high frequency of severe cases presenting on hospital admission is related and possibly a consequence of the fragmented and precarious health care system of Peru. In addition, the lack of resources such as general and ICU beds [15,33], and the low health expenditure during the last few decades [4,33,34], lead to high rates of self-medication and out-of-hospital care [14]. The socioeconomic inequalities in our country are reflected in the large proportion of vulnerable people and informal workers [5,35,36], who were unable to comply with the lockdown regulations imposed by the government due to economic dependence on daily earnings [33]. In addition to all of these factors, national and health sector corruption has disrupted the implementation of public health policies due to successive changes of government and health ministers [4,37].

The serious limitations of hospital beds at medical and ICU wards might ensure only the worst patients were triaged for admission to our hospital. This particular condition of Peruvian health system cannot be compared with other countries with better resources, and is one of the main reasons that a high level of oxygen demand was an independent variable related to mortality. Also, our severe compromised study population could set aside a large number of well-described related factors, although age, an independent risk factor remains significant in our sample.

Our results confirm that low oxygen saturation is a relevant predictor of mortality, even independently of some socio-demographic characteristics, medical background and other clinical findings. Low oxygen saturation measured by pulse oximetry has demonstrated to be useful for early prediction of outcomes, monitoring, and guiding hospitalization in other scenarios [38–40]. The presence of low oxygen saturation in patients requiring high flow oxygen delivery has been demonstrated to be more reliable than the remaining signs and symptoms for predicting clinical deterioration during the course of COVID-19 [41] and is a predictor of mortality associated with severe disease. This simple test should be assessed more often in clinical evaluations and regular check-ups among COVID-19 patients in resource-limited countries.

Even the multivariate analysis did not show it, our high mortality rate mainly reflects the low number of intensive care unit beds, 5 per 100,000 inhabitants; an insufficient offer for the great number of critically affected patients [4]. To reduce this mortality, Peru has recently inverted in a massive vaccination program, increase the number of ICU beds, with mechanical ventilators, monitors and trained nurses and doctors, and the implementation of temporary oxygen centers [42]; scarce supplies revealing decades-long of poor investment in public health [4].

Some other important findings merit consideration. About 98% of the patients included in our study received antibiotics during their hospitalization. Other countries have documented rates of antibiotic use of up to 74% [43] attributed to the lack of information about COVID-19 management at the beginning of the pandemic, the concomitant elevation of infectious markers [44–46] and the well described “compassionate” use [47–50]. The use of antibiotics during hospitalization could be explained by the confusion of a negative test in a patient with pneumonia, considering that the majority of the test made were serological rapid test with a limited value in diagnosis of acute COVID-19 infection. In addition, self-medication rates before admission to the hospital, a demonstrated harmful practice [14,51,52] were very high in our study (28.9%), being corticosteroids and antibiotics the most frequently self-administered.

Self-medication as a pre-hospital treatment was mainly self-prescribed and motivated by fear, anxiety and supported by early studies, expert opinion and media misinformation [4]. Some patients use antibiotics alone, some corticosteroids alone, and some a mix of them, probably with other unproved medication. Certainly, during the first wave, the lack of knowledge and fear among the population led to the overuse of medications that were wrongfully prescribed and promoted by the press and social media [47]. In fact, this situation was a consequence of the lack of assistance in primary health care centers, which were closed at the beginning of the pandemic and during the first wave [53].

Finally, we found no association between mortality and corticosteroid use during hospitalization. This may be due to the use of high doses of methylprednisolone and hydrocortisone at the beginning of the pandemic until the benefits of dexamethasone were demonstrated in patients with moderate to severe COVID-19 pneumonia [54]. However, the main explanation for this finding is the shortage of ICU beds, since the poor clinical status and high oxygen requirement levels on admission indicated the need for critical care in most of our patients [4].

Limitations of our study are related to the retrospective nature of data collection, which may have led to certain biases in data registration and collection. A minor proportion of patients (2.5%) had the opportunity to made a PCR-RT diagnostic test, but the majority of patients without had a clinical correlate with COVID-19 and the test used to classify them was the serological rapid-test, the only available test in Peruvian setting. Furthermore, our study could not include the majority of admitted patients due to the limited access to medical records. However, this study was performed in a national hospital with a large and representative study population, using the national electronic death information system and obtaining results that are consistent with other publications.

## Conclusions

In-hospital mortality among patients with COVID-19 in Peru is high. Advanced age and high oxygen requirement on hospital admission are associated with greater mortality. Our findings are largely due to the low-quality health care system in our country.

## Supporting information

S1 Table Confirmed COVID-19 deaths per million people, case-fatality ratio and GDP per capita by country on August 2020. (DOCX)

S2 Table Oxygen requirement levels based on three parameters. (DOCX)

S3 Table Characteristics associated with oxygen requirement levels. (DOCX)

S1 Graph Confirmed COVID-19 deaths per million vs GPD per capita on August 2020. (PDF)

S1 File (DTA)

## References

[1] National Health Commission of the People’s Republic of China. Update on the novel coronavirus pneumonia outbreak. (http://www.nhc.gov.cn/xcs/yqtb/202002/18546da875d74445bb537ab014e7a1c6.shtml [Accessed 16 Mar 2021]) 2021.

[2] World Health Organization. A public health emergency of international concern over the global outbreak of novel coronavirus declared by WHO. (https://www.who.int/emergencies/diseases/novel-coronavirus-2019/interactive-timeline [Accessed 16 Mar 2021]) 2021.

[3] COVID-19 Dashboard by the Center for Systems Science and Engineering (CSSE) at Johns Hopkins University (JHU). (https://coronavirus.jhu.edu/data/mortality. [Accessed 16 Jun 2021]) 2021.

[4] Schwalb A, Seas C. The COVID-19 Pandemic in Peru: What Went Wrong? Am J Trop Med Hyg 2021;104: 1176–8. doi: 10.4269/ajtmh.20-1323 33591940 PMC8045664

[5] Taylor L. Covid-19: Why Peru suffers from one of the highest excess death rates in the world. BMJ 2021;372: n611. doi: 10.1136/bmj.n611 33687923

[6] Li B, Yang J, Zhao F, et al. Prevalence and impact of cardiovascular metabolic diseases on COVID-19 in China. Clin Res Cardiol 2020;109: 531–8. doi: 10.1007/s00392-020-01626-9 32161990 PMC7087935

[7] Feng Y, Ling Y, Bai T, Zhi L, Wang X, Liu L, et al. COVID-19 with Different Severities: A Multicenter Study of Clinical Features. Am J Respir Crit Care Med 2020;201: 1380–8. doi: 10.1164/rccm.202002-0445OC 32275452 PMC7258639

[8] Yang W, Cao Q, Qin L, Wang X, Cheng Z, Pan A, et al. Clinical characteristics and imaging manifestations of the 2019 novel coronavirus disease (COVID-19): A multi-center study in Wenzhou city, Zhejiang, China. J Infect 2020;80: 388–93. doi: 10.1016/j.jinf.2020.02.016 32112884 PMC7102539

[9] Gue YX, Tennyson M, Gao J, Ren S, Kanji R, Gong DA. Development of a novel risk score to predict mortality in patients admitted to hospital with COVID-19. Sci Rep 2020;10: 21379. doi: 10.1038/s41598-020-78505-w 33288840 PMC7721695

[10] Kamran SM, Mirza ZE, Moeed HA, Naseem A, Hussain M, Fazal I, et al. CALL Score and RAS Score as Predictive Models for Coronavirus Disease 2019. Cureus 2020;12: e11368. doi: 10.7759/cureus.11368 33304701 PMC7721080

[11] Ji D, Zhang D, Xu J, Chen Z, Yang T, Zhao P, et al. Prediction for Progression Risk in Patients With COVID-19 Pneumonia: The CALL Score. Clin Infect Dis 2020;71: 1393–9. doi: 10.1093/cid/ciaa414 32271369 PMC7184473

[12] Mejía F, Medina C, Cornejo E, Morello E, Vásquez S, Alave J, et al. Oxygen saturation as a predictor of mortality in hospitalized adult patients with COVID-19 in a public hospital in Lima, Peru. PLoS One 2020;15: e0244171. doi: 10.1371/journal.pone.0244171 33370364 PMC7769479

[13] Vidal-Anzardo M, Solis G, Solari L, Minaya G, Ayala-Quintanilla B, Astete-Cornejo J, et al. Evaluation of a rapid serological test for detection of IgM and IgG antibodies against SARS-CoV-2 under field conditions. Rev Peru Med Exp Salud Publica 2020;37: 203–9. doi: 10.17843/rpmesp.2020.372.5534 32876207

[14] Quispe-Cañari JF, Fidel-Rosales E, Manrique D, Mascaró-Zan J, Huamán-Castillón KM, Chamorro-Espinoza SE, et al. Self-medication practices during the COVID-19 pandemic among the adult population in Peru: A cross-sectional survey. Saudi Pharm J 2021;29: 1–11. doi: 10.1016/j.jsps.2020.12.001 33519270 PMC7832015

[15] Montenegro-Idrogo JJ, Chiappe-González AJ. Decentralized budget execution and COVID-19 lethality in Peru. Rev Peru Med Exp Salud Publica 2020;37: 781–2. doi: 10.17843/rpmesp.2020.374.5786 33566925

[16] Abate SM, Checkol YA, Mantefardo B. Global prevalence and determinants of mortality among patients with COVID-19: A systematic review and meta-analysis. Ann Med Surg (Lond) 2021;64: 102204. doi: 10.1016/j.amsu.2021.102204 33692899 PMC7931690

[17] Hospital Nacional Arzobispo Loayza. (http://hospitalloayza.gob.pe/files/TRAS_e8b60d764cd3ec5_pdf [Accessed 18 Mar 2021]) 2021.

[18] Ministerio de Salud. Lima-Perú. MINSA. (https://www.gob.pe/institucion/hospitalloayza/noticias/185141-hospital-loayza-realiza-nueva-ampliacion-para-hospitalizacion-de-pacientes-con-covid-19 [Accessed 18 Mar 2021]) 2021.

[19] Chemical and Engineering News (CEN). Developing countries face diagnostic challenges as the COVID-19 pandemic surges. (https://cen.acs.org/analytical-chemistry/diagnostics/Developing-countries-face-diagnostic-challenges/98/i27 [Accessed 11 Feb 2022]) 2020.

[20] Ministerio de Salud. Lima-Perú. MINSA. Documento técnico: Manejo ambulatorio de personas afectadas por COVID-19 en el Perú (https://cdn.www.gob.pe/uploads/document/file/830595/RM_375-2020-MINSA.PDF [Accessed 11 Feb 2022]) 2020.

[21] Instituto de Evaluación de Tecnologías en Salud e Investigación. Guía de Práctica Clínica para el Manejo de COVID-19: Guía en Versión Extensa. Versión 2, julio 2021. Lima: EsSalud; 2021. (https://iris.paho.org/handle/10665.2/53894 [Accessed 22 Oct 2021]) 2021.

[22] Brown SM, Grissom CK, Moss M, Rice TW, Schoenfeld D, Hou PC, et al. Nonlinear Imputation of Pao2/Fio2 From Spo2/Fio2 Among Patients with Acute Respiratory Distress Syndrome. Chest 2016;150: 307–13. doi: 10.1016/j.chest.2016.01.003 26836924 PMC4980543

[23] Sanz F, Dean N, Dickerson J, Jones B, Knox D, Fernández-Fabrellas E, et al. Accuracy of PaO2 /FiO2 calculated from SpO2 for severity assessment in ED patients with pneumonia. Respirology 2015;20: 813–8. doi: 10.1111/resp.12560 25998684

[24] Ministerio de Salud. Sistema Nacional de Defunciones (SINADEF), Lima, Perú. (https://www.minsa.gob.pe/defunciones/ [Accesed 6 Feb 2021]) 2021.

[25] Bellan M, Patti G, Hayden E, Azzolina D, Pirisi M, Acquaviva A, et al. Fatality rate and predictors of mortality in an Italian cohort of hospitalized COVID-19 patients. Sci Rep 2020;10: 20731. doi: 10.1038/s41598-020-77698-4 33244144 PMC7692524

[26] Noor FM, Islam MM. Prevalence and Associated Risk Factors of Mortality Among COVID-19 Patients: A Meta-Analysis. J Community Health. 2020 Dec;45(6):1270–1282. doi: 10.1007/s10900-020-00920-x 32918645 PMC7486583

[27] Open COVID—Perú. (https://opencovid-peru.com [Accessed 16 Jun 2021]) 2021.

[28] Videnza Consultores. The Peruvian health system. figshare. Figure. (10.6084/m9.figshare.14977839.v1. [Accessed 30 Oct 2021]) 2021.

[29] The Associated Press. Scarce medical oxygen worldwide leaves many gasping for life. (https://apnews.com/article/ebola-virus-health-conakry-ap-top-news-virus-outbreak-df97326ec00fb7cc4abf5b3821ace984 [Accessed 11 Feb 2022]) 2020.

[30] HelpPeru. The oxygen crisis in Peru. (https://www.help-peru.org/the-oxygen-campaign/ [Accessed 11 Feb 2022]) 2021.

[31] Khan MMA, Khan MN, Mustagir MG, Rana J, Islam MS, Kabir MI. Effects of underlying morbidities on the occurrence of deaths in COVID-19 patients: A systematic review and meta-analysis. J Glob Health 2020;10: 020503. doi: 10.7189/jogh.10.020503 33110586 PMC7567434

[32] Chauhan A, Kaur R, Chakrbarti P, Pal A. "*Silent Hypoxemia*" Leads to Vicious Cycle of Infection, Coagulopathy and Cytokine Storm in COVID-19: Can Prophylactic Oxygen Therapy Prevent It? Indian J Clin Biochem 2021: 1–5. 10.1007/s12291-021-00967-0 PMC7958103 33746377

[33] Rees GH, Peralta-Quispe F, Scotter C. The implications of COVID-19 for health workforce planning and policy: the case of Peru. Int J Health Plann Manage 2021;19: 1–8. doi: 10.1002/hpm.3127 33604953 PMC8013434

[34] Miller MJ, Loaiza JR, Takyar A, Gilman RH. COVID-19 in Latin America: Novel transmission dynamics for a global pandemic? PLoS Negl Trop Dis. 2020;14: e0008265. doi: 10.1371/journal.pntd.0008265 32379757 PMC7205198

[35] Rocha R, Atun R, Massuda A, Rache B, Spinola P, Nunes L, et al. Effect of socioeconomic inequalities and vulnerabilities on health-system preparedness and response to COVID-19 in Brazil: a comprehensive analysis. Lancet Glob Health 2021;9: e782–92. doi: 10.1016/S2214-109X(21)00081-4 33857500 PMC8041360

[36] Martinez-Valle A. Public health matters: why is Latin America struggling in addressing the pandemic? J Public Health Policy 2021;42: 27–40. doi: 10.1057/s41271-020-00269-4 33510400 PMC7841039

[37] García PJ. Corruption in global health: the open secret. Lancet. 2019;394: 2119–24. doi: 10.1016/S0140-6736(19)32527-9 31785827

[38] Chua F, Vancheeswaran R, Draper A, Vaghela T, Knight M, Mogal R, et al. Early prognostication of COVID-19 to guide hospitalisation versus outpatient monitoring using a point-of-test risk prediction score. Thorax 2021;0: 1–8. doi: 10.1136/thoraxjnl-2020-216425 33692174

[39] Booth A, Reed AB, Ponzo S, Yassaee A, Aral M, Plans D, et al. Population risk factors for severe disease and mortality in COVID-19: A global systematic review and meta-analysis. PLoS One 2021;16: e0247461. doi: 10.1371/journal.pone.0247461 33661992 PMC7932512

[40] Lopez-Pais J, Otero DL, Ferreiro TG, Antonio CEC, Muiños PJA, Perez-Poza M, et al. Fast track triage for COVID-19 based on a population study: The soda score. Prev Med Rep 2020;21: 101298. doi: 10.1016/j.pmedr.2020.101298 33489725 PMC7809432

[41] Chatterjee NA, Jensen PN, Harris AW, Nguyen DD, Huang HD, Cheng RK, et al. Admission respiratory status predicts mortality in COVID- 19. Influenza Other Respi Viruses. 2021;00: 1–4. doi: 10.1111/irv.12869 34028169 PMC8242415

[42] Agencia Peruana de Noticias. Health Min: Peru is preparing to face third COVID-19 wave. (https://andina.pe/ingles/noticia-health-min-peru-is-preparing-to-face-third-covid19-wave-857093.aspx [Accessed 11 Feb 2022] 2021.

[43] Chedid M, Waked R, Haddad E, Chetata N, Saliba G, Choucair J. Antibiotics in treatment of COVID-19 complications: a review of frequency, indications, and efficacy. J Infect Public Health 2021;14: 570–6. doi: 10.1016/j.jiph.2021.02.001 33848886 PMC7870433

[44] Fabre V, Karaba S, Amoah J, Robinson M, Jones G, Dzintars K, et al. The Role of Procalcitonin in Antibiotic Decision-Making in Covid-19 Infection. Infect Control Hosp Epidemiol 2021: 1–24. 10.1017/ice.2021.175 PMC8485015 33866995

[45] Staub MB, Beaulieu RM, Graves J, Nelson GE. Changes in antimicrobial utilization during the coronavirus disease 2019 (COVID-19) pandemic after implementation of a multispecialty clinical guidance team. Infect Control Hosp Epidemiol 2020: 1–7. doi: 10.1017/ice.2020.1291 33100250 PMC7683821

[46] Ryu S, Hwang Y, Ali ST, Kim DS, Klein EY, Lau EHY, et al. Decreased use of broad-spectrum antibiotics during COVID-19 epidemic in South Korea. J Infect Dis 2021;jiab208. doi: 10.1093/infdis/jiab208 33856455 PMC8083342

[47] Melo JRR, Duarte EC, Moraes MV, Fleck K, Arrais PSD. Self-medication and indiscriminate use of medicines during the COVID-19 pandemic. Cad Saude Publica 2021;37(4): e00053221. doi: 10.1590/0102-311X00053221 33852694

[48] Zhang A, Hobman EV, De Barro P, Young A, Carter DJ, Byrne Ml. Self-Medication with Antibiotics for Protection against COVID-19: The Role of Psychological Distress, Knowledge of, and Experiences with Antibiotics. Antibiotics (Basel) 2021;10: 232. 33668953 10.3390/antibiotics10030232 PMC7996601

[49] Chong WH, Saha BK, Ananthakrishnan R, Chopra A. State-of-the-art review of secondary pulmonary infections in patients with COVID-19 pneumonia. Infection 2021: 1–15. doi: 10.1007/s15010-021-01602-z 33709380 PMC7951131

[50] Baskaran V, Lawrence H, Lansbury LE, Webb K, Safavi S, Zainuddin NI, et al. Co-infection in critically ill patients with COVID-19: an observational cohort study from England. J Med Microbiol 2021;70. doi: 10.1099/jmm.0.001350 33861190 PMC8289210

[51] Gras M, Gras-Champel V, Moragny J, Delaunay P, Laugier D, Masmoudi K, et al. Impact of the COVID-19 outbreak on the reporting of adverse drug reactions associated with self-medication. Ann Pharm Fr 2021: S0003-4509. doi: 10.1016/j.pharma.2021.02.003 33631179 PMC7899020

[52] Tandon T, Dubey AK, Dubey S, Arora E, Hasan MN. Effects of COVID-19 pandemic lockdown on medical advice seeking and medication practices of home-bound non-COVID patients. J Educ Health Promot 2021;10: 28. doi: 10.4103/jehp.jehp_481_20 33688537 PMC7933611

[53] Herrera-Añazco P, Uyen-Cateriano A, Mezones-Holguin E, Taype-Rondan A, Mayta-Tristan P, Malaga G, et al. Some lessons that Peru did not learn before the second wave of COVID-19. Int J Health Plann Manage 2021: 10. 10.1002/hpm.3135 PMC8014877 33595137

[54] RECOVERY Collaborative Group, Horby P, Lim WS, Emberson JR, Mafham M, Bell JL, et al. Dexamethasone in Hospitalized Patients with Covid-19. N Engl J Med 2021;384: 693–704. doi: 10.1056/NEJMoa2021436 32678530 PMC7383595

