# Supplementary material for: High mortality among hospitalized adult patients with COVID-19 pneumonia in Peru: A single centre retrospective cohort study
Source: PLoS One. 2022 Mar 8;17(3):e0265089. doi: 10.1371/journal.pone.0265089 (PMC8903290; doi:10.1371/journal.pone.0265089)
Supplement: S1 Table — (DOCX) [file pone.0265089.s001.docx]

| **Country** | **Deaths per million (n)** | **Case-fatality ratio* (%)** | **GDP per capita ($)** |
| --- | --- | --- | --- |
| Peru | 2206.63 | 6.2 | 12,237 |
| Italy | 587.78 | 1.3 | 35,220 |
| Brazil | 568.33 | 2.4 | 14,103 |
| United States | 550.84 | 1.2 | 54,225 |
| Mexico | 494.5 | 6.0 | 17,336 |
| Colombia | 383.53 | 2.3 | 13,255 |
| Argentina | 189.89 | 1.4 | 18,934 |
| Romania | 189.31 | 2.5 | 23,313 |
| Poland | 53.95 | 2.1 | 27,216 |
| Ukraine | 59.93 | 2.4 | 7,894 |

GDP: Gross domestic product

*number of deaths either per 100 confirmed cases.

Source: COVID-19 Data Repository by the Center for Systems Science and Engineering (CSSE) at Johns Hopkins University. Available in: <https://github.com/CSSEGISandData/COVID-19>
