# Supplementary material for: High mortality among hospitalized adult patients with COVID-19 pneumonia in Peru: A single centre retrospective cohort study
Source: PLoS One. 2022 Mar 8;17(3):e0265089. doi: 10.1371/journal.pone.0265089 (PMC8903290; doi:10.1371/journal.pone.0265089)
Supplement: S2 Table — (DOCX) [file pone.0265089.s002.docx]

|  | Level 1 | Level 2 | Level 3 |
| --- | --- | --- | --- |
| PaFiO2 ratio | >300 | 150 a 300 | <150 |
| Oxygen delivery | 0.21 a 0.28 | 0.21 a 0.4 | > 0.4 |
| SO2 at FiO2 0.21 | >90 % | 80-90% | <80% |
