# Supplementary material for: High mortality among hospitalized adult patients with COVID-19 pneumonia in Peru: A single centre retrospective cohort study
Source: PLoS One. 2022 Mar 8;17(3):e0265089. doi: 10.1371/journal.pone.0265089 (PMC8903290; doi:10.1371/journal.pone.0265089)
Supplement: S3 Table — (DOCX) [file pone.0265089.s003.docx]

| **Variables** | **Level 1**  **n=194** | **Level 2**  **n=643** | **Level 3**  **n= 681** | **P value** |
| --- | --- | --- | --- | --- |
| Age in years^a^ | 54.5 ± 14.9 | 58.7 ± 14.0 | 63.1 ± 13.6 | < 0.001 |
| Corticosteroid use during hospitalization | 144 (74.2) | 569 (88.5) | 626 (91.9) | <0.001 |
| Leukocytes ≥ 10000 (per mm3) | 79 (40.7) | 342 (53.2) | 436 (64.1) | <0.001 |
| Neutrophils ≥ 7500 (per mm3) | 90 (46.4) | 402 (62.5) | 489 (71.8) | <0.001 |
| Lymphocytes < 1000 (per mm3) | 88 (45.4) | 342 (53.2) | 383 (56.2) | 0.004 |
| D-dimer ≥ 1.0 (ug/ml) | 8 (4.1) | 66 (10.3) | 105 (15.4) | <0.001 |
| Lactate dehydrogenase ≥ 350 (U/L) | 52 (26.8) | 316 (49.1) | 473 (69.5) | <0.001 |
| C-reactive protein ≥ 7.5 (mg/L) | 78 (40.2) | 374 (58.2) | 411 (60.4) | <0.001 |
| Lactate ≥ 2 (mmol/L) | 26 (13.4) | 83 (12.9) | 126 (18.5) | 0.012 |
| Alanine aminotransferase ≥ 35 (UI/L) | 106 (54.6) | 383 (59.6) | 377 (55.4) | 0.032 |
| Albumin < 3.5 (mg/dl) | 36 (18.6) | 140 (21.8) | 223 (32.8) | <0.001 |
| Ferritin ≥ 800 (ug/L) | 30 (15.5) | 161 (25.1) | 198 (29.1) | <0.001 |
| Mortality | 45 (23.2) | 213 (33.1) | 501 (73.6) | <0.001 |
| Values are in number and percentage (%) |  |  |  |  |
| ^a^Mean ± standard deviation |  |  |  |  |
|  |  |  |  |  |
|  |  |  |  |  |
