# Supplementary figures and images for: High mortality among hospitalized adult patients with COVID-19 pneumonia in Peru: A single centre retrospective cohort study
Source: PLoS One. 2022 Mar 8;17(3):e0265089. doi: 10.1371/journal.pone.0265089 (PMC8903290; doi:10.1371/journal.pone.0265089)

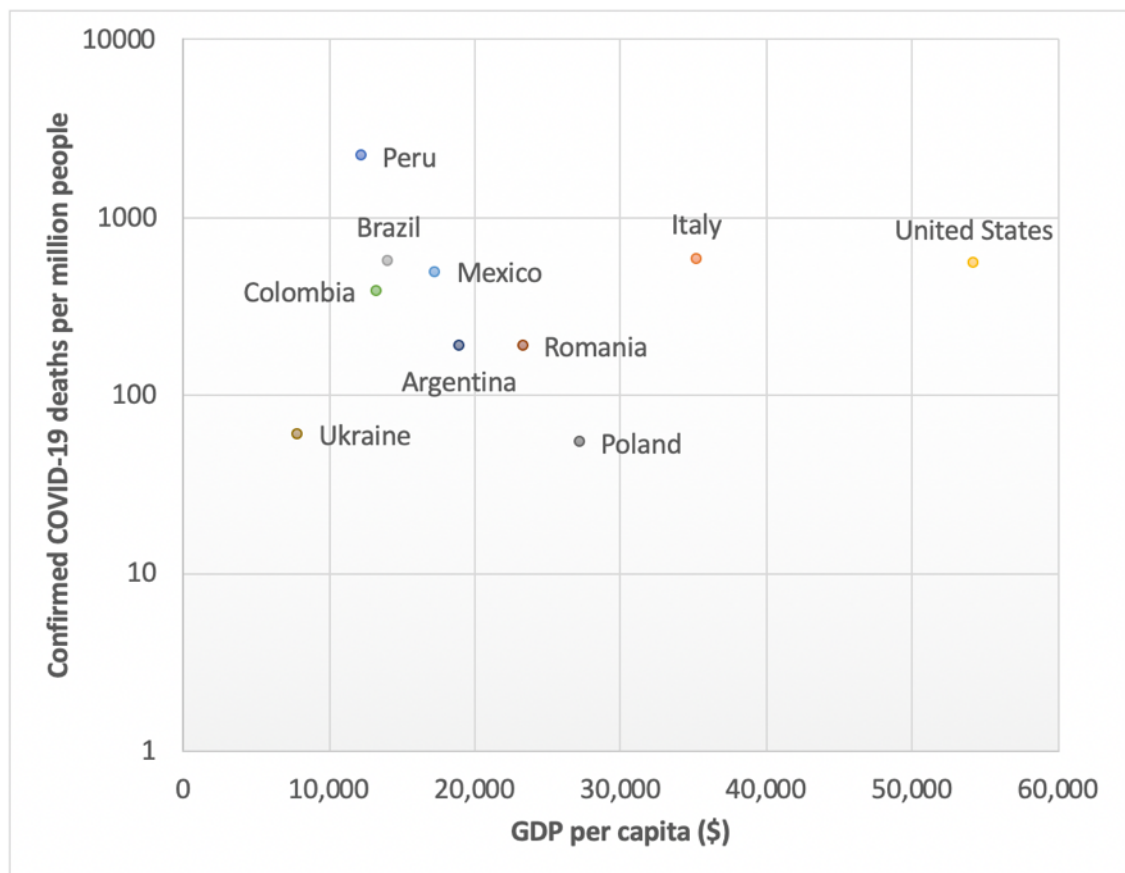

Supplement: S1 Graph — (PDF) [file pone.0265089.s004.pdf]
